# Oleuropein-Enriched Extract From Olive Mill Leaves by Homogenizer-Assisted Extraction and Its Antioxidant and Antiglycating Activities

**DOI:** 10.3389/fnut.2022.895070

**Published:** 2022-06-23

**Authors:** Katherine Márquez, Nicole Márquez, Felipe Ávila, Nadia Cruz, Alberto Burgos-Edwards, Ximena Pardo, Basilio Carrasco

**Affiliations:** ^1^Centro de Estudios en Alimentos Procesados (CEAP), CONICYT-Regional, Gore Maule R0912001, Talca, Chile; ^2^Escuela de Nutrición y Dietética, Facultad de Ciencias de la Salud, Universidad de Talca, Talca, Chile

**Keywords:** olive leaf extract (OLE), oleuropein (OLEU), antioxidant activity (AA), antiglycating effect, homogenizer-assisted extraction

## Abstract

Olive oil consumption has increased in the last two decades and consequently, its wastes have increased, which generates a tremendous environmental impact. Among the by-products are the olive mill leaves, which are easier and inexpensive to treat than other olive by-products. However, little research has been done on their chemical composition and potential bioactivity. Hence, in this study, olive mill leaves were used to obtain Oleuropein-Enriched Extracts (OLEU-EE) using Conventional Extraction, Ultrasound-Assisted Extraction, and Homogenization-Assisted Extraction. These three techniques were evaluated using a Factorial Design to determine the parameters to obtain an OLEU-EE with high contents of Total Phenolic Compounds (TPC), Antioxidant Activity (AA), and Oleuropein concentration (OLEU). From the results, the Homogenizer-Assisted Extraction (HAE) technique was selected at 18,000 rpm, solid:liquid ratio 1:10, and 30 s of homogenization with 70% ethanol, due to its high TPC (5,196 mg GA/100 g), AA (57,867 μmol of TE/100 g), and OLEU (4,345 mg of OLEU/100 g). In addition, the antiglycating effect of OLEU-EE on the levels of (1) fluorescent Advanced Glycation End Products (AGEs) were IC_50_ of 0.1899 and 0.1697 mg/mL for ^1^λ_EXC_ 325/λ_EM_ 440 and ^2^λ_EXC_ 389/λ_EM_ 443, respectively; (2) protein oxidative damage markers such as dityrosine (DiTyr), N-formylkynurenine (N-formyl Kyn), and kynurenine (Kyn) were IC_50_ of 0.1852, 0.2044, and 0.1720 mg/mL, respectively. In conclusion, OLEU-EE from olive mill leaves has different capacities to inhibit AGEs evidenced by the IC_50_ of fluorescent AGEs and protein oxidation products, together with the scavenging free radical evidenced by the concentration of Trolox Equivalent. Therefore, OLEU-EE could be potential functional ingredients that prevent oxidative damage caused by free radicals and AGEs accumulation.

## Introduction

There is a growing demand for the consumption of healthy foods for their nutritional and functional contribution. In this way, olive oil consumption has increased in the last two decades, due to its beneficial effects on human health (1, 2). This increase has generated a positive economic impact on olive oil production worldwide (3, 4). However, the traditional extraction method yields about 20% of olive oil and about 80% corresponds to by-products, which generates an enormous environmental impact (3, 5, 6). A two-phase system made it possible to save water and energy in the 1990s. However, by-products are still generated and generally used for direct combustion, fresh animal feed, and fertilization in the field (7, 8). Little or no treatment of these residues generates a great environmental impact, due to their high content of organic matter, phytotoxic compounds, and pH (6, 9). These by-products include olive pomace, olive mill wastewater, and olive leaves (2, 10).

Olive leaves can be obtained directly from the olive tree and it is known as “pruning leaves,” and from the pruning of an olive tree, around 25 kg of by-products are produced between branches and leaves (11). Also, olive leaves can be obtained from the olive mill in the process of cleaning the fruit from the separated olive branches using a blowing machine and it is known as “olive mill leaves” (12). Olive mill leaves contain soil, leaves, and small branches that represent approximately 10% of the fruit weight (4, 6, 13). The last by-product is more economical than pruning leaves and safer than alperujo to use as a functional food additive (high content of organic matter and phytotoxics). Although, there is little research on its chemical composition and potential bioactivity (12). Olive Leaf Extract (OLE) can be obtained from olive leaves, with reported properties such as antioxidant, anti-inflammatory, anti-hypertensive, antimicrobial, hypoglycemic, hypolipidemic, and anticholesterolemic (4, 6, 14–16). Bioactive properties are generally associated with its phenolic compounds (14, 16, 17). The main phenolic compounds present in olive leaves are oleuropein (OLEU), luteolin-7-glucoside, apigenin-7-glucoside, verbascoside, tyrosol, and hydroxytyrosol (HYT) (14, 18). In this study, Oleuropein-Enriched Extracts (OLEU-EE) were obtained and optimized, with OLEU being the major phenolic in olive leaves (18, 19).

Currently, there are several reports on the valorization of olive oil by-products. However, implementing them on an industrial scale is technically expensive and complex (20). Industries are challenged to use new technologies that utilize cost-efficient and sustainable processes (21). The green techniques with low environmental impact and high efficiency were used in this research (22, 23). Among the green extraction methods are Ultrasound-Assisted Extraction (UAE) and Homogenizer-Assisted Extraction (HAE). Ultrasounds have a cavitation effect that accelerates heat and mass transfer by altering the cell walls of plants, allowing a more significant release of micronutrients (24) and reducing solvent extraction time (21). UAE has been applied to several natural sources for the extraction of phenolics (21), including olive leaves (25, 26).

High-speed homogenization is a technique that involves rotating the sample at high speed, which is introduced axially into the dispersion head and then forced radially through slots in the rotor arrangement. High accelerations produce extreme shear and thrust forces. Another advantage of HAE is the reduced volume of solvent, low energy cost, and reduced processing time, since in a few seconds, the size of the plant material particles decreases, thus promoting the release of compounds into the medium (22, 27). HAE has been used successfully to extract phenolics from natural sources (22, 28). HAE has only been applied to olive leaves reported by Yücel et al. (23). Although, to our knowledge, HAE has not been applied to olive mill leaves using several solvents. In addition, HAE has been used in the delignification of plant biomass, which could further benefit the extraction of phenolics by breaking down the plant fiber and releasing these bioactive compounds (29). It has been reported that the extraction process affects the chemical composition of the extracts and consequently their potential bioactivity (30). Therefore, the extraction conditions of the total phenolic compounds, and in particular of OLEU, were evaluated using a Design of Experiments (DOE) screening using food grade solvents such as ethanol/water (8). In addition, the antioxidant activity of OLEU-EE was determined as a response.

On the other hand, modern diets are based on thermally processed foods which generate one of the main chemical modifications in proteins during food processing or theMaillard reaction (31). Consequently, high consumption of thermally processed foods increases Advanced Glycation End Products (AGEs) (32). AGEs are a complex and heterogeneous group of compounds whose accumulation contributes to increased oxidative stress inflammation and are related to numerous chronic non-communicable diseases (NCDs) and the aging process (31, 33). OLEU has important reported properties such as antioxidant, anti-inflammatory, antimicrobial, and hypolipidemic activity (30, 34–37). However, there is little information regarding the antiglycating effect of OLEU-EE (30), and some articles attribute the antiglycating effect to the HYT of extracts enriched in this compound (36, 38). In this way, obtaining AGEs inhibitors from the valorization of Olive mill leaves could have beneficial effects in reducing NCDs. That is why, in this research, the antiglycant effect of OLEU-EE on AGE levels and oxidative modifications of proteins were evaluated.

Therefore, this research aims to determine the extraction conditions that allow obtaining OLEU-EE in a high concentration of OLEU, total polyphenols, and antioxidant activity, from olive mill leaves using cost-efficient techniques. In addition, the selected OLEU-EE was evaluated for its anti-glycation effect demonstrating the action of OLEU-EE on oxidative stress mediated by radicals and AGEs.

## Materials and Methods

### Chemicals and Standards

The solvent used was ethanol supplied by Merck Millipore (Darmstadt, Germany), and the reagents trolox, gallic acid, 2,2-diphenyl−1-picrylhydrazyl (DPPH), acetonitrile, phosphoric acid, oleuropein, 3-hydroxytyrosol, aminoguanidine, D(+)-glucose, bovine serum albumin (defatted fraction), and sodium azide were supplied by Sigma Aldrich (St. Louis, MO, USA).

### Sampling

Olive mill leaves, “Arbequina” (*O. europaea*), obtained directly from the blowing machine of the oil mill of “Olivos de Talca” in Huilliborgoa (Maule Region, Chile) were used. The samples were washed with tap water in a 1:10 ratio twice for 10 min with mechanical agitation at 600 rpm. Excess water was drained off and dried in an oven at 105°C until reaching humidity between 2 and 5%. Samples are ground using the Multifunction Grinder (Power Mix, Sindelen) using the 2:10 min program. Then, they are sieved to a particle size of 425 μm (mesh 40, CISA Barcelona, Spain). Samples are stored in the dark at 4°C.

### Extraction of Phenolic Compounds

The extraction of total phenols and especially oleuropein from olive mill leaves was evaluated using a factorial DOE that considered three independent variables and two to three combined levels, which was carried out with the software Modde v.7.0—Umetrics. For each variable, the levels are represented by a lower (−1), middle (0), and upper (+1) level, respectively. The values of these levels were chosen according to the literature based on olive leaves or other plant matrices (22, 23, 26). The independent variables were extraction technique (CE, HAE, and UAE), solid:liquid ratio (1:5 and 1:10), and time as a function of technique (30 and 45 s for HAE; 30 and 60 min for AUE; and 24 and 48 h for CE). The response variables were Oleuropein concentration (mg OLEU/100 g), total polyphenols (mg GA/100 g), and antioxidant activity of OLEU-EE (μmol TE/100 g). The design required 12 experiments with three replicates.

Ethanolic extracts were obtained from dried, ground, and sieved (mesh #40) olive mill leaves. In brief, 10 g of powdered leaf were mixed with 50 or 100 mL of 80% ethanol as extractant depending on the DOE experiment number, according to Table 1. The three techniques for extraction are described in continuation.

**Table 1:** DOE factorial for the optimization of the extraction of total phenolics and especially oleuropein from olive mill leaves.

**#**	**Factorial DOE**			**Description of variables**		
	**Time**	**s:l ratio**	**Technique**	**Time**	**s:l ratio**	**Technique**
1	−1	1	0	30 s	1:10	HAE
2	−1	−1	−1	24 h	1:5	CE
3	1	−1	0	45 s	1:5	HAE
4	−1	−1	0	30 s	1:5	HAE
5	−1	1	−1	24 h	1:10	CE
6	−1	1	1	30 min	1:10	UAE
7	1	−1	−1	48 h	1:5	CE
8	1	−1	1	60 min	1:5	UAE
9	−1	−1	1	30 min	1:5	UAE
10	1	1	−1	48 h	1:10	CE
11	1	1	0	45 s	1:10	HAE
12	1	1	1	60 min	1:10	UAE

### Conventional Extraction (CE)

Solid-liquid CE consisted of the solubilization of phenolic compounds using 80% ethanol. The sieved olive mill leaves and the solvent were placed in an amber flask on a stirring plate at 1,000 rpm and room temperature. The variables were the solid:liquid ratio and stirring time (Table 1).

### Homogenizer-Assisted Extraction (HAE)

Solid-liquid HAE consisted of solubilization of phenolic compounds using 80% ethanol. The sieved olive mill leaves together with the solvent were placed in a tall beaker; the solubilization is carried out with the Ultraturrax homogenizer (T 25 easy-clean control EC C S000 IKA, Germany) to 18,000 rpm. The equipment has a sensor that allows permanent control of the temperature of the medium, which was maintained between 35 and 40°C, and in some cases using a water-ice bath. The variables were the solid:liquid ratio and the homogenization time three times at 10-s intervals (Table 1).

### Ultrasound-Assisted Extraction (UAE)

Solid-liquid UAE consisted of solubilization of phenolic compounds using 80% ethanol. The sieved olive mill leaves together with the solvent were placed in a beaker and the probe-type ultrasonic and probe temperature were introduced. The parameters of the Sonicator (ultrasonic processor Q125 and probe CL−18, Qsonica) pulsed 30 s on/30 s off and 40% power rate. The equipment has a temperature probe with an alarm that allows permanent control of the temperature of the medium, which was maintained between 35 and 40°C, and in some cases using an ice-water bath. The variables were the solid:liquid ratio and elapsed time (Table 1).

### Total Phenol Content (TPC) Assay

TPC assays were determined using the 96-well microplate spectrophotometric method based on Folin–Ciocalteu slightly modified literature method (39), using a multimode microplate reader (Synergy HTX Biotex, USA). In brief, 30 microliters of previously diluted samples were introduced into the micro-wells and 30 microliters of 10% Folin–Ciocalteau Reagent are added. After 2 min, 240 microliters of 5.0% (w/v) Na_2_CO_3_ are added. The plate is incorporated into a microplate spectrophotometer reader (BioTek) and the samples are incubated at 37°C for 30 min until their measurement at absorbance of 760 nm. The gallic acid calibration curve (0.5–12 mg/L) was elaborated similarly. The results were expressed as mg GA/100 g of extract. Data were presented as the average of triplicate analyses.

### DPPH Radical Scavenging Activity Assay

DPPH assays were determined using the 96-well microplate spectrophotometric method based on the slightly modified literature method (40), using a multimode microplate reader (Synergy HTX Biotex, USA). In brief, 20 microliters of previously diluted samples were introduced into the micro-wells and 180 microliters of DPPH 150 μmol/L diluted in methanol are added. The plate is incorporated into a microplate spectrophotometer reader (BioTek), and the samples are incubated at 37°C for 30 min until their measurement at absorbance of 517 nm. The trolox calibration curve (0.36–36 μmol/L) was elaborated in the same manner. The results were expressed as μmol TE/100 g of extract. Data were presented as the average of triplicate analyses.

### HPLC-DAD Determination of Principal Phenolic Compounds

Phenolic quantification of ethanolic OLEU-EE was performed using a method previously described by Bouaziz and Sayadi et al. (13). A YL9100 Plus HPLC (Young IN Chromass, Korea), YL9160 PDA Detector (Young IN Chromass, Korea), YL9150 Plus LC Autosampler (Young IN Chromass, Korea), YL9131 Column Compartment (Young IN Chromass, Korea), and a Spherisorb ODS2 C−18 column (250 × 4.6 mm id, 5 μm) (Waters, USA) were used. Elution was performed at a flow rate of 0.6 mL/min using a mixture of 0.1% phosphoric acid (mobile phase A) and acetonitrile/water 70:30 v/v (mobile phase B) as the mobile phase; flow of 0.6 ml/min. The solvent gradient is as follows: from 90% solvent A in 0–10 min to 75% in 25 min, to 20% in 35 min, to 0% in 40 min, and to 90% solvent A in 50 min. Phenolic quantification was performed at 280 nm using Chromatography Data System (YL-Clarity).

### Identification of Phenolic Compound in OLEU-EE by HPLC-MS/MS

Samples were analyzed through UHPLC-MS/MS in a Waters Acquity Chromatographer (Waters, Milford, CT, USA). The detection system consisted of an Acquity PDA eλ detector and a Xevo TQD QqQ-MS mass spectrometer with an electrospray ionization (ESI) source, both connected in tandem. The column ACQUITY UPLC^®^ BEH C18 (1.7 μm; 2.1 × 150 mm) (Waters, Milford, CT, USA) was employed for the separation of compounds. System control, peak detection, and data acquisition were performed with the Masslynx V4.1 software.

Chromatographic separation was performed using a linear gradient solvent system consisting of water (solvent A) and acetonitrile (solvent B), both with formic acid 0.1% at a flow rate of 0.3 mL/min. The gradient elution program was as follows: 0–6.25 min, 7.00–17.50% B; 6.25–8.75 min, 17.50–56.00% B; 8.75–9.25 min, 56.00–70.00% B; 9.25–10.0 min, 70.00–100% B; 10.0–12.5 min, 100–7.00% B, and 12.5–13.0 min with 7.00% B. The samples were dissolved in a mixture of acetonitrile and water (1:1) at 5 mg/mL and filtered through 0.22 μm nylon syringe filters for injection. The injection volume was 10 μL and the temperature of the column was 40°C.

The UV spectra from 200 to 500 nm were acquired for peak characterization. The triple quadrupole mass spectrometer (QqQ) was operated in negative ion mode, employing MS scan mode within a range of m/z 100 to 700 with 0.15 s of scan time. Instrument operating parameters were as follows: source temperature, 150°C; capillary voltage, 4.0 kV; desolvation temperature, 500°C; desolvation gas flow, 900 L/h; cone gas flow, 50 L/h; cone voltage, 25 V; collision energy, 20 V. Nitrogen was employed as cone and desolvation gas, while the collision gas was argon.

### Inhibition of AGEs and Protein Oxidation Using OLEU-EE

The inhibitory effect of OLEU-EE, OLEU (pure compound), HYT (pure compound), and aminoguanidine (AMIG, standard) on glycation and oxidation of some amino acids were assessed using fluorescence spectroscopy. Bovine serum albumin (10 mg/mL) was incubated with glucose (0.5 M) and OLEU-EE at the following concentrations: 0, 0.05, 0.1, 0.25, 0.5, 1, and 2.5 mg/mL. Samples were prepared in phosphate buffer of 0.1 mol/L, using sodium azide 0.2 mg/mL as an antimicrobial agent and incubated for 40 h, at 55°C. Fluorescence measurements were carried out by diluting the incubated mixtures six times with buffer phosphate at 100 mM pH 7.4, giving a total volume of 3 mL. Posteriorly, fluorescence was measured according to the following excitation and emission wavelengths; AGEs at λ_EXC_/λ_EM_ = 325/440 (41) and λ_EXC_/λ_EM_ = 389/443 (42); Protein oxidation products at λ_EXC_/λ_EM_ = 330/415 for dityrosine (DiTyr), λ_EXC_/λ_EM_ = 365/480 nm for kynurenine (Kyn), and at λ_EXC_/λ_EM_ = 325/434 nm for N-formylkynurenine (N-formyl Kyn). Fluorescence analyses were carried out in a Perkin Elmer LS 55 fluorescence spectrofluorometer (PerkinElmer Ltd., Waltham, MA, USA).

The inhibition percentage (% Inhibition) of AGEs and protein oxidation products was calculated using the following expression:


(1) $$\begin{array}{r} \%\;Inhibition=\left(1-\frac{BSA_{Glu/P}-BSA_{P}}{BSA_{Glu}}\right)\times 100 \end{array}$$

BSA_Glu/P_ represents BSA incubated with glucose and extracts (extracts, pure compounds, or AMIG); BSA_P_ corresponds to serum albumin incubated with extracts, pure compounds, or AMIG at the same concentration as BSA_Glu/P_ and BSA_Glu_ corresponds to serum albumin incubated with glucose.

### Statistical Analyses

All statistical analyzes were developed using only the GraphPad Prism v7 program. Statistical differences between the means of different samples were determined by one-way analysis of variance (ANOVA) followed by the Tukey's HSD test (detailed in the Supplementary Table S1).

## Results and Discussion

### Total Phenolic Content of the OLEU-EE

TPC in OLEU-EE was determined by Folin–Ciocalteu assay adapted to microwells. From the results obtained, its content in the extracts ranges from 2,270 to 5,196 mg GA/100 g extract. Compared with the literature, Sahin and Samli and del Mar Contreras et al. (12, 26) reported TPC in ethanolic OLE ranges from 518 to 2,037 mg GA/100 g and 2,100 to 13,100 mg GA/100 g using UAE in both types of research. On the other hand, Herrero et al. (8) reported TPC in ethanolic and aqueous extracts of OLE in the range from 2,830 to 5,870 mg GA/100 g using Pressurized Liquid Extraction (PLE). Yücel et al. (23) reported TPC in OLE with several solvents in the range from 782 to 4,740 mg GA/100 g using HAE. From the research mentioned earlier, only del Mar Contreras et al. (12) indicate that olive mill leaves were used; however, the other studies are based on pruning leaves or do not specify.

**Figure 1:**
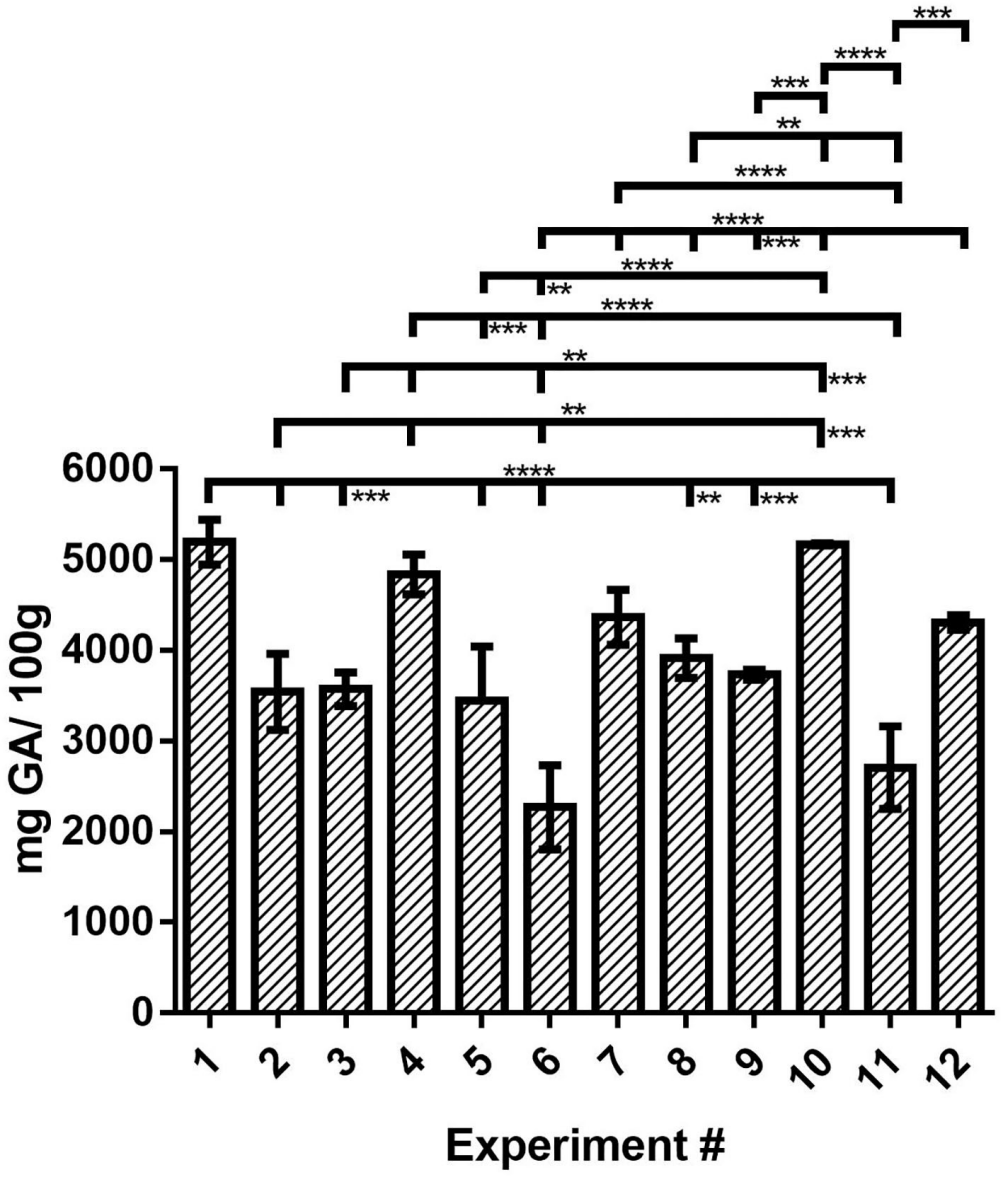
TPC of the OLEU-EE determined by Folin–Ciocalteu assay and comparison of mean ± SD values by one-way ANOVA with Tukey's test of the TPC for the 12 experiments. With a confidence interval of 95% (α = 0.05), significance is represented by brackets with two asterisks (**) identify adjusted P-values between 0.01 and 0.001, three asterisks (***) identify adjusted P-values between 0.001 and 0.0001, and four asterisks (****) identify adjusted *P*-values < 0.0001.

From Figure 1, if TPC is compared only on the based-on extraction technique, the highest TPC content is obtained using HAE and CE, and then UAE. Furthermore, analyzing the results of the lowest level (−1) of the extraction times (24 h, 30 s, and 30 min for CE, HAE, and EAU, respectively), the s:l ratio does not influence the TPC content (not significant (ns); *p* ≥ 0.05) for all three techniques with this time level (−1). In the case of the highest level (+1) of extraction times (48 h, 45 s, and 60 min for CE, HAE, and UAE, respectively), for the three techniques, the s:l ratio does not influence the TPC content (ns). Therefore, the highest TPC of the mill leaves was obtained with the HAE [experiments #1 and #4 (ns)] and CE [experiments #10; #1 and #10 (ns), and #10 and #4 (ns)] techniques.

Of these three conditions, those of experiment #1 or #4 are chosen since this profitable process is carried out in only 30 s of homogenization. Statistical differences between the means of different samples for TPC were determined by one-way analysis of variance (ANOVA) followed by the Tukey test using the GraphPad Prism v7 program (Supplementary Table S1).

### Principal Phenolic Compounds by HPLC-DAD

OLEU was identified in all the OLEU-EE of this study (Supplementary Figures S1, S2), and for quantification, a calibration curve was constructed with a high purity OLEU standard and whose equation of the line is described in Equation (2) with a coefficient of determination of 0.997. In contrast, HYT was identified only in some samples but was not quantified because it was below the limit of quantification of the method (results not shown). The other phenolic compounds were not identified at this stage of the study, since the objective is to obtain extracts with a high content of OLEU as the main response to be evaluated. From the results obtained, its content in the extracts ranges from 1225 to 4345 mg OLEU/100 g extract. Compared with the literature, del Mar Contreras et al. (12) reported a concentration of OLEU in ethanolic OLEU in the range from 1,100 to 2,900 mg OLEU/100 g extract using UAE as a green technology.


(2) $$\begin{array}{r} Area={29,388}^{*}OLEU\left(\frac{mg}{L}\right)+\;555 \end{array}$$

**Figure 2:**
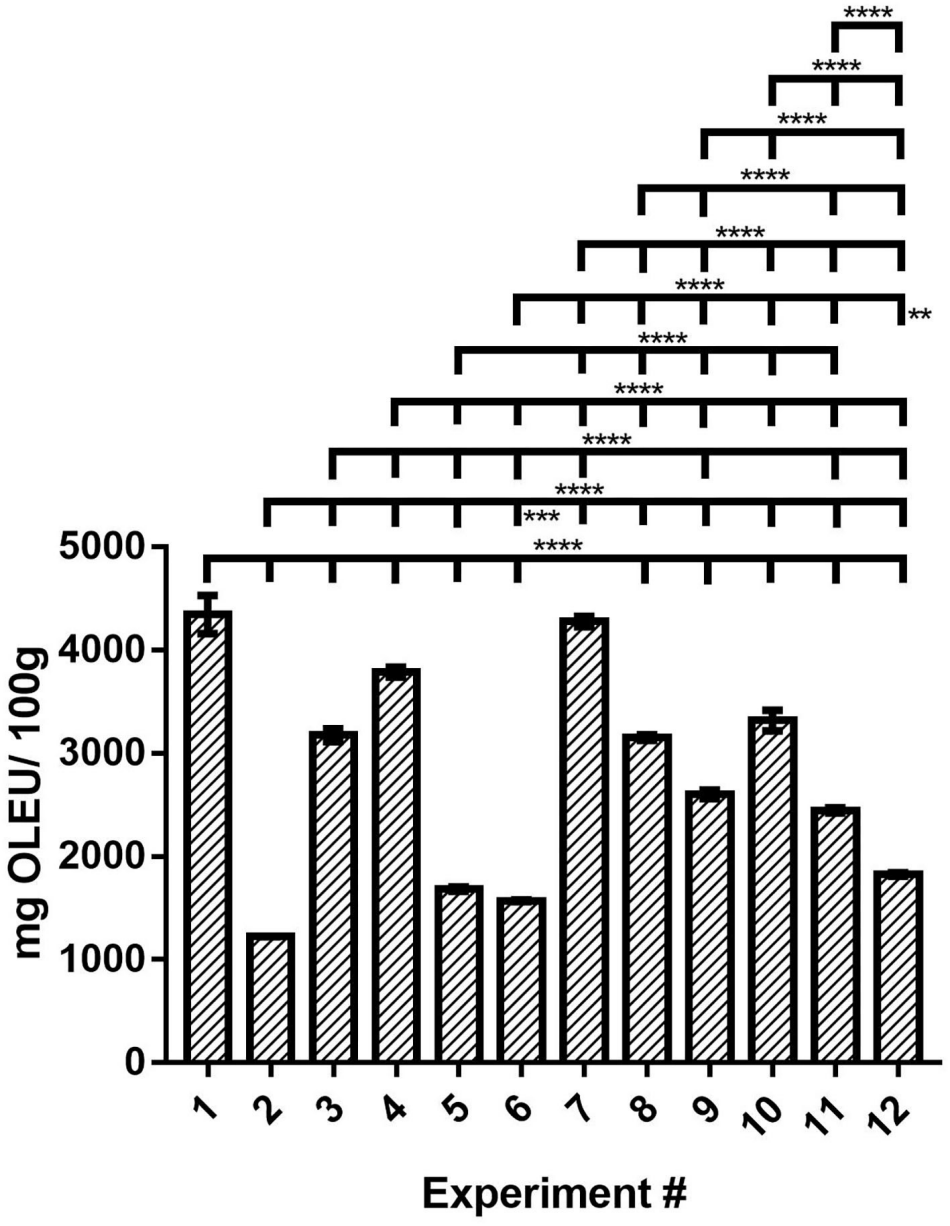
OLEU concentration of OLEU-EE quantified by HPLC-DAD and comparison of mean ± SD values by one-way ANOVA with Tukey's test of the OLEU content for the 12 experiments. With a confidence interval of 95% (α = 0.05), significance is represented by brackets with two asterisks (**) identify adjusted *P-*values between 0.01 and 0.001, three asterisks (***) identify adjusted *P-*values between 0.001 and 0.0001, and four asterisks (****) identify adjusted *P-*values < 0.0001.

From Figure 2, if OLEU concentration is compared only bases on extraction technique, the highest OLEU concentration is obtained using HAE and CE, and then UAE. Moreover, analyzing the results for the lowest level (−1) of extraction times (24 h, 30 s, and 30 min for CE, HAE, and UAE, respectively), as the s:l ratio increases from 1:5 to 1:10 the OLEU concentration increases when using CE and HAE techniques (^****^; extremely significant), except in the case of UAE where the s:l ratio is inversely proportional to OLEU concentration (^***^; extremely significant). On the contrary, in the case of the highest level (+1) of extraction times (48 h, 45 s, and 60 min for CE, HAE, and UAE, respectively), as the s:l ratio increases from 1:5 to 1:10, the OLEU concentration decreases when all three techniques are used (^****^). Therefore, the highest OLEU concentration from olive mill leaves was obtained with the HAE (experiment #1) and CE (experiment #7) techniques (#1 and #7 ns). Of these two conditions, those of experiment #1 is chosen since this cost-efficient process is carried out in only 30 s of homogenization versus 48 h of agitation. Statistical differences between the means of different samples for OLEU concentration were determined by one-way analysis of variance (ANOVA) followed by the Tukey test using the GraphPad Prism v7 program (Supplementary Table S1).

### Antioxidant Activity (AA) Assays in Microplates

AA in OLEU-EE was determined by DPPH radical scavenging activity assay adapted to microwells (40). From the results obtained, its content in the extracts ranges from 29,348 to 61,381 μmol TE/100 g extract. Compared with the literature, Zuntar et al. (43) reported AA by DPPH assay in ethanolic and aqueous extracts of OLE in the range from 24,780 to 32,710 μmol TE/100 g using high-voltage electrical discharges (HVED) as a green technology. On the other hand, Yücel et al. (23) reported AA by DPPH assay in OLE with several solvents in the range from 11,460 to 32,720 μmol TE/100 g extract using HAE. In the aforementioned investigations, the antioxidant activity by DPPH in olive leaves was evaluated, but not in olive mill leaves.

**Figure 3:**
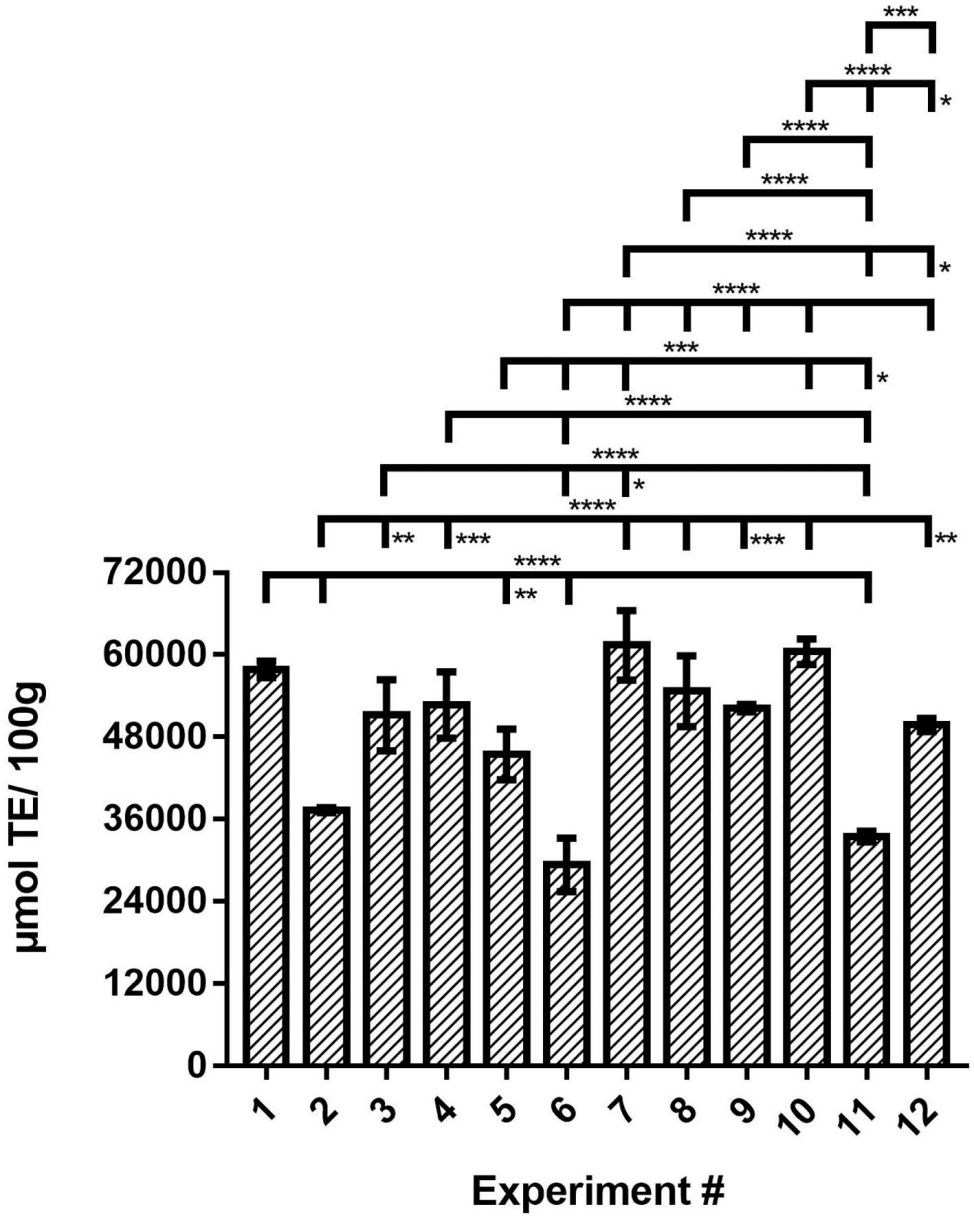
AA of the OLEU-EE determined by DPPH radical scavenging activity assay and comparison of mean ± SD values by one-way ANOVA with Tukey's test of the AA for the 12 experiments. With a confidence interval of 95% (α = 0.05), significance is represented by brackets with: one asterisk (*) identify adjusted *P-*values between 0.01 and 0.05, two asterisks (**) identify adjusted *P-*values between 0.01 and 0.001, three asterisks (***) identify adjusted *P-*values between 0.001 and 0.0001, and four asterisks (****) identify adjusted *P-*values < 0.0001.

From the results in Figure 3, if AA is compared only based on extraction technique, the highest radical scavenging activity is obtained using CE and HAE, and then UAE. Moreover, analyzing the results for the lowest level (−1) of extraction times (24 h, 30 s, and 30 min for CE, HAE, and UAE, respectively), as the s:l ratio increases from 1:5 to 1:10, the AA decreases when using the UAE technique (^****^), for the CE and HAE techniques, the s:l ratio does not influence the AA (ns) for this time level (−1). On the contrary, in the case of the highest level (+1) of extraction times (48 h, 45 s, and 60 min for CE, HAE, and UAE, respectively), as the s:l increases from 1:5 to 1:10, the AA decreases when using the HAE technique (^****^), in the case of UAE and CE, there is no significant effect of s:l ratio on the AA for this level (ns). Therefore, the highest AA in OLE from olive mill leaves was obtained with the CE technique with 48 h of agitation and 1:5 or 1:10 ratio (Experiment #7 and #10 ns) and the HAE technique with 1:10 ratio and 30 s of homogenization at 18,000 rpm (Experiment #1; #1 and #7 ns; #1 and #10 ns). The conditions of experiment #1 are chosen since this cost-efficient process is carried out in only 30 s of homogenization instead of 48 h of agitation. Statistical differences between the means of different samples for AA were determined by one-way analysis of variance (ANOVA) followed by the Tukey test using the GraphPad Prism v7 program (Supplementary Table S1 in Supplementary Material).

### Factorial Design of Experiments

From the results of DOE Factorial of three factors and two or three variables (Full Fac -Mixed Interaction), the factors time and s:l ratio were defined as the quantitative type and the factor technique as the qualitative type. The first two factors were evaluated with two levels (−1 to +1) and the technique factor was evaluated with three levels (HAE, CE, and UAE). The DOE's were adjusted with the Partial Least Squares (PLS) method. The DOE's for evaluating TPC, OLEU, and AA in the OLEU-EE are listed below in Table 2.

**Table 2:** DOE's to evaluate the effect of time, s:l ratio, and extraction technique on the responses TPC, OLEU concentration, and AA by DPPH assay.

**Response**	**Method PLS**	**Coefficients**		
**TPC**	•2 Components •R_2_: 0.825 •Q_2_: 0.641 •RSD: 380 •N: 32 •Rep.: 0.862 •Outliers: 1c, 11c, 1b, 6c.	3,946*Cte + 205,1*Time−38,1*s:l (ns) – 196,5*(Time*s:l)		
		**Tech: HAE**	**Tech: CE**	**Tech: UAE**
		Tech: 7,95 (ns) Time*Tech: −684 s:l*Tech: −79,0 (ns)	Tech: 148 (ns) Time*Tech: 410 s:l*Tech: 170	Tech: −156 (ns) Time*Tech: 274 s:l*Tech: −90,5 (ns)
				
**OLEU**	•3 Components •R_2_:0.985 •Q_2_: 0.932 •RSD: 161 •N: 32 •Rep.: 0.998 •Outliers: 1a, 9c, 8c, 6a.	2,822*Cte + 313.3*Time −263.4*s:l −291.0*(Time*s:l)		
		**Tech: HAE**	**Tech: CE**	**Tech: UAE**
		Tech: 457.1 Time*Tech: −755.4 s:l*Tech: 114.3	Tech: -84.4 Time*Tech: −771.5 s:l *Tech: 129.2	Tech:−372.8 Time*Tech: −16 (ns) s:l*Tech: −243.5
				
**AA**	•2 Components •R_2_: 0.854 •Q_2_: 0.634 •RSD: 4921 •N: 32 •Rep.: 0.929 •Outlier: 3a, 8c, 1c, 8b.	48,820*Cte + 4,209*Time −3,249*s:l −1,413*(Time*s:l:) (ns)		
		**Tech: HAE**	**Tech: CE**	**Tech: UAE**
		Tech: −897.7 (ns) Time*Tech: −9,214 s:l *Tech: −1,593 (ns)	Tech: 1,809.5 (ns) Time*Tech: 5,691 s:l*Tech: 2,690	Tech: −912.7 (ns) Time*Tech: 3,523 s:l*Tech: −4,283
				

*Rep., Reproducibility; s:l, s:l ratio; Tech, Technique; ns, no significant*.

From the TPC response polynomial, the coefficients that contribute significantly to the TPC are time, s:l, and the interactions Time^*^s:l and Time^*^Tech. Of these four coefficients, the most relevant is the time^*^Tech interaction for the three techniques. The non-significant factors would be the s:l and Tech, although they cannot be excluded from the model because their interactions are significant. From the OLEU response polynomial, all the coefficients contribute significantly to the response, both individually and in their interactions (except the interaction time^*^Tech UAE). Finally, from the AA response polynomial, the coefficients that contribute significantly to the response are time, s:l, Time^*^Tech, and s:l^*^Tech (except the interaction s:l^*^Tech UAE).

**Figure 4:**
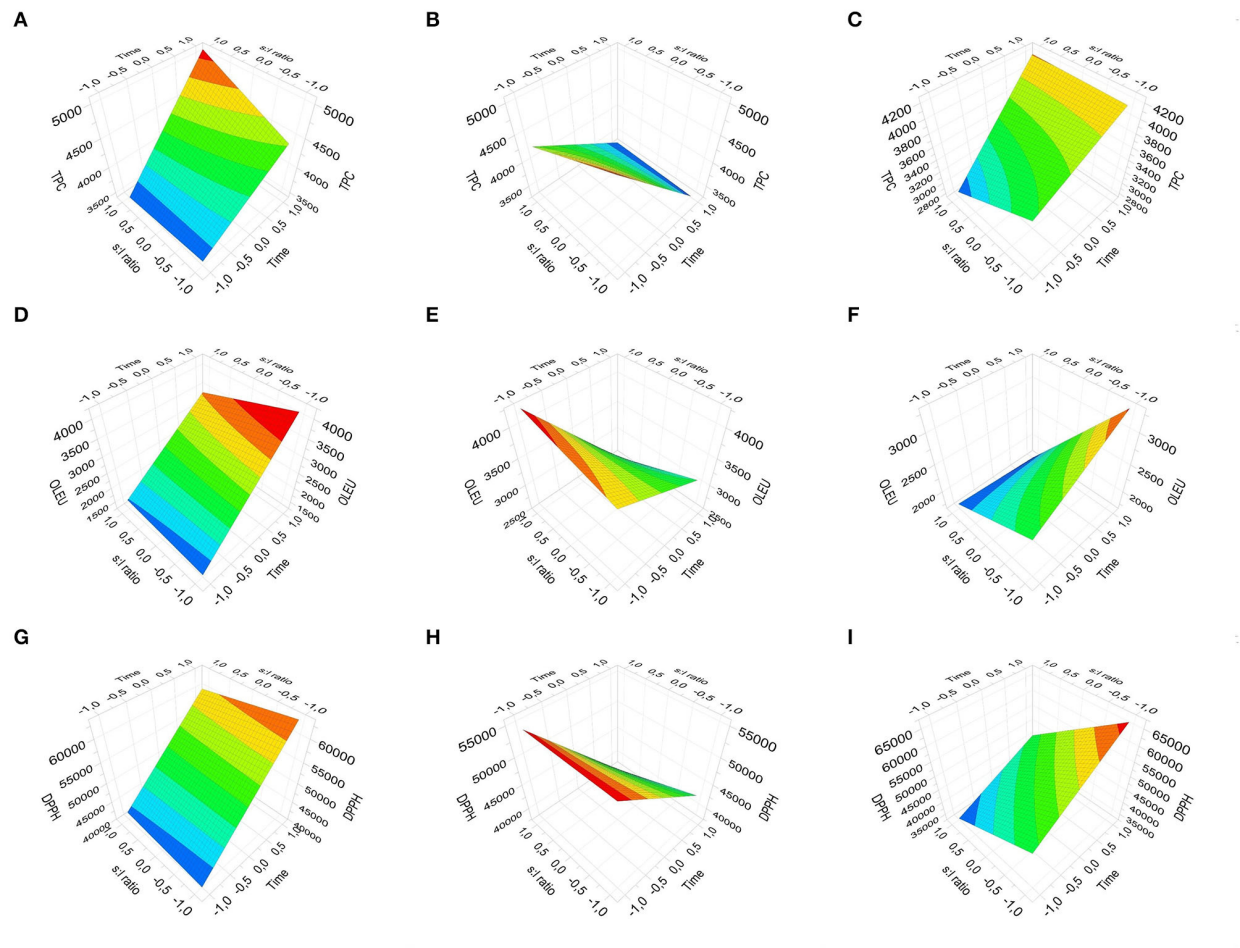
Response Surface Plot for the three responses: TPC by CE, HAE, and UAE **(A–C)**, OLEU concentration by CE, HAE, and UAE **(D–F)**, and DPPH assay by CE, HAE, and UAE **(G–I)**.

From Figure 4, analyzing the response surfaces, TPC by CE (Figure 4A), the higher response is obtained at a longer time and higher s:l ratio. For TPC by HAE (Figure 4B), the highest response is obtained at a shorter time and independent of the s:l ratio. For TPC by UAE (Figure 4C), the highest response is obtained at a longer time and independent of the s:l ratio. For OLEU by CE (Figure 4D), a higher response is obtained at a longer time and a lower s:l ratio. For OLEU by HAE (Figure 4E), a higher response is obtained at a shorter time and a higher s:l ratio. For OLEU by UAE (Figure 4F), the highest response is obtained at a longer time and lower s:l ratio. For DPPH by CE (Figure 4G), the highest response is obtained at a longer time and lower s:l ratio. For DPPH by HAE (Figure 4H), the highest response is obtained at a shorter time and independent of the s:l ratio. For DPPH by UAE (Figure 4I), the highest response is obtained at a longer time and lower s:l ratio. It should be noted that, although it is not the objective of this research, to optimize the TPC, OLEU, and AA responses, it would be necessary to add levels since a plateau is not observed in the response surface graphs. Except in HAE, which is close to the response optimal.

In summary, OLEU-EE obtained with the conditions of DOE experiment #1 was selected because it obtained the highest concentration of OLEU, TPC, and high AA, with a cost-efficient process. Although in some instances, the results are similar to the conventional technique, the processing time is prioritized, which would be reduced from 48 h of agitation to only 30 s of homogenization at high speed and shearing.

### Phenolic Compounds in OLEU-EE by UHPLC-MS

The OLEU-EE selected from the DOE (OLEU-EE #1) was analyzed by UHPLC-MS to tentatively identify oleuropein and its derivatives, as well as other phenolic compounds that could be present in a lower concentration. The UHPLC-MS analyses allowed the tentative identification of 20 compounds in the OLEU-EE from olive mill leaves. Under our experimental conditions, 10 secoiridoids, 5 flavonoids, 3 cinnamic acid derivatives, 1 organic acid, and 1 simple phenol were detected. The compounds were assigned based on their retention time, UV spectra, and masses of the ions compared to the literature. The spectrometric data of the assigned compounds are shown in Table 3 and the selected ion chromatograms of each peak are depicted in Supplementary Figures S3, S4.

**Table 3:** Tentative identification of the components of the OLEU-EE from olive mill leaves.

**Peak**	**Rt (min)**	**UVmax**	**[M-H]^**−**^**	**MS fragments**	**Tentative identification**
1	1.52		389.60	227.14	Oleoside
2	1.55		191.24		Quinic acid
3	1.62		179.20	135.83	Caffeic acid derivative
4	4.16–4.26	220, 280	153.09		HYT
5	6.17		389.61	226.34, 209.67	Sologanoside isomer
6	8.06–8.19		377.21	307.11, 275.72	OLEU aglycone isomer 1
7	9.27–9.47	220, 280	525.77	209.31, 195.27, 165.21	Demethyl-OLEU
8	9.35–9.55	267, 355	609.79	300.55	Rutin
9	9.45–9.65		623.55	460.67	Verbascoside (acteoside)
10	9.46–9.66	267, 345	593.40	285.3	Luteolin rutinoside
11	9.51–9.71	260, 341	447.39	284.96, 241.06	Luteolin hexoside isomer 1
12	9.82–9.96	268, 340	447.63	284.80	Luteolin hexoside isomer 2
13	10.05–10.25	223, 280	539.49	377.37, 227.22	OLEU
14	10.25		637.60	623.64, 461.78	Methyl verbascoside
15	10.52	223, 280	553.49	538.65	Methyl OLEU
16	10.71	345	285.32	255.33, 267.24, 212.95, 177.36	Luteolin
17	11.30		377.81	195.27	OLEU aglycone isomer 2
18	11.77		361.41	328.59	Ligstroside aglycone
19	11.87		377.81	345.3, 195.08, 165.25	OLEU aglycone isomer 3
20	12.49		377.81	274.20	OLEU aglycone isomer 4

The only organic acid detected was quinic acid **(2)**, tentatively identified by the [M-H]- ion at m/z 191 (44). Similarly, one simple phenol was observed, the HYT **(4)**, tentatively assigned due to its [M-H]- ion at m/z 153 (44). Three cinnamic acid derivatives were observed in the OLEU-EE. The first **(3)** showed two ions at m/z 179 and 135, suggesting a caffeic acid molecule (45). Considering its retention time, it was tentatively assigned as a caffeic acid derivative **(3)**. The other two compounds **(9** and **14)** showed ions at m/z 623 and 461, characteristics of verbascoside/acteoside (44, 45). The last **(14)** also showed an MS ion at m/z 637, suggesting one methyl (14 amu) more than the verbascoside ion. Therefore, compounds **9** and **14** were tentatively identified as verbascoside and methyl verbascoside, respectively.

Among the flavonoids, four sugar-bonded and one aglycone were detected. The only quercetin derivative **(8)** was tentatively identified as rutin, considering the MS ions at m/z 609 and 301(44, 46). Compounds **10, 11**, and **12** showed the ion at m/z 285, in agreement with a luteolin core. The other ion at m/z 593 observed for compound **10**, suggested an attached rutinose (308 amu) to the luteolin Aglycon, whereas the ion at m/z 447 indicates a bonded hexose (162 amu). Therefore, compounds **10**, **11**, and **12** were tentatively identified as luteolin rutinoside **(10)** and its hexoses 1 **(11)** and 2 **(12)**, respectively. Peak **16** showed the [M-H]- ion at m/z 285, in agreement with luteolin aglycon and the diagnostic ions at m/z 267, 255, 213, and 177 (45, 47). Thus, compound **16** was tentatively assigned as luteolin.

As expected, a variety of secoiridoids were detected in the extract. Compounds **1** and **5** showed a pseudomolecular ion at m/z 389. Both also exhibited an ion at m/z 227, in agreement with oleoside/secologanoside aglycon, after a neutral loss of one hexose moiety (162 amu). Further, the last **(5)** also showed one ion at m/z 209, suggesting the oleoside/secologanoside aglycon after a neutral loss of one water molecule (18 amu) (46). Considering their retention time, compounds **1** and **5** were tentatively assigned as oleoside and secologanoside, respectively. Peaks **6, 17, 19**, and **20** were tentatively assigned as OLEU aglycon isomers, based on their [M-H]- ion at m/z 377 (46). The characteristic ions at m/z 345, 307, and 275 observed for peaks **6, 19**, and **20** supported the assignation (46). Further, the ion at m/z 195 was detected for peaks **17** and **19** and is also compatible with OLEU aglycon-related compounds (45, 48). Compound **7** showed a pseudomolecular ion at m/z 525 and MS ions at m/z 209, 195, and 165, in agreement with a demethyloleuropein (46). Thus, it was tentatively identified as such. Peak **13** was assigned as OLEU due to its characteristic [M-H]- ion at m/z 539 and the ions at m/z 377 and 227 (45, 46). The identity of compound **13** was further confirmed by comparison with a commercial standard. Compound **15** showed a pseudomolecular ion at m/z 553, compatible with methyl-OLEU. It also showed a loss of a methyl group (14 amu) leading to the ion at m/z 539, compatible with an OLEU moiety (46). Thus, compound **15** was tentatively identified as methyl-OLEU. Finally, compound **18** was assigned as ligstroside aglycon due to the presence of the diagnostic ions at m/z 361 and 329 (46). All of these compounds were previously reported in *O. europaea* leaves supporting our results (44–46).

### Antiglycating Effects of OLEU-EE in Proteins

**Figure 5:**
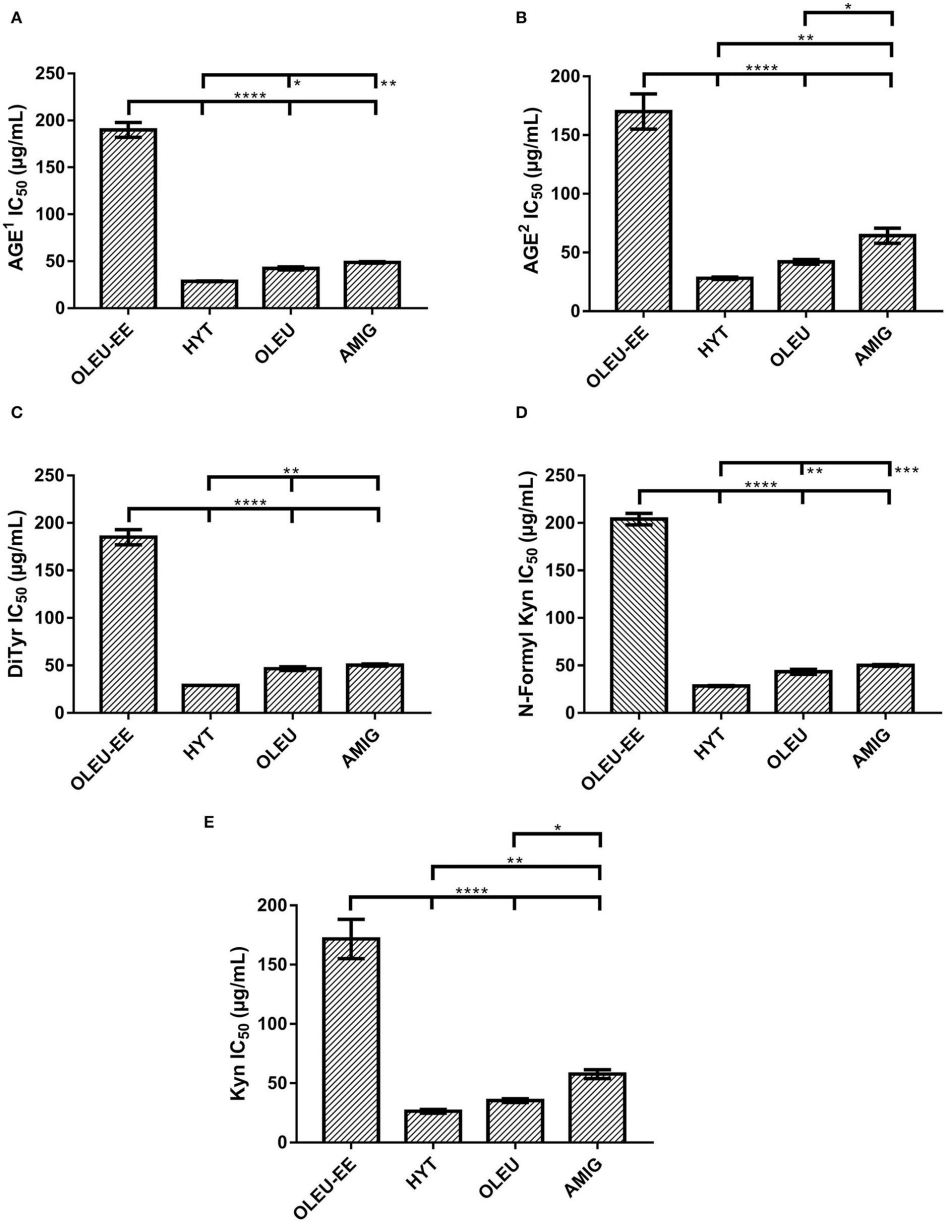
Effect of OLEU-EE #1 obtained from olive mill leaves in the inhibition of fluorescent **(A)** AGEs^1^ (^1^λ_EXC_ 325/λ_EM_ 440); **(B)** AGEs^2^ (^2^λ_EXC_ 389/λ_EM_ 443); and protein oxidative modifications produced by the incubation of BSA with glucose; **(C)** DiTyr; **(D)** N-Formyl Kyn; and **(E)** Kyn. The means ± SD values of OLEU-EE with HYT, OLEU, and AMIG were compared by one-way ANOVA with Tukey's test. With a confidence interval of 95% (α = 0.05), significance is represented by brackets with one asterisk (*) identify adjusted *P-*values between 0.01 and 0.05, two asterisks (**) identify adjusted *P-*values between 0.01 and 0.001, three asterisks (***) identify adjusted *P-*values between 0.001 and 0.0001, and four asterisks (****) identify adjusted *P-*values < 0.0001.

The effect of the OLEU-EE #1 on the inhibition of fluorescent AGEs and oxidative modifications of proteins induced by glucose was evaluated. Thus, anti-glycating effect of OLEU-EE #1 on the levels of AGEs and oxidated amino acids (DiTyr), N-formyl Kyn, and Kyn were quantified in proteins using fluorescence. IC_50_ was calculated for each protein modification and compared to AMIG, OLEU, and HYT (pure compounds). These results are shown in Figure 5 and statistical differences between the means of different samples were determined by one-way analysis of variance (ANOVA) followed by the Tukey test (Supplementary Table S2).

Navarro et al. (36) and Koch (49) have evaluated the activity against the generation of AGEs mediated by OLEU-EE and extracts enriched in hydroxytyrosol (HYT-EE), reporting a decrease in the AGEs of BSA generated using glucose, glyoxal, and methylglyoxal as glycating agents and using the formation of fluorescent protein-bound AGEs, respectively. IC_50_ values of 0.716 mg/mL and 0.294 mg/mL have been reported for OLEU-EE and HYT-EE, respectively (36). Also, IC_50_ values of 2.14 mg/mL and 0.500 mg/mL have been reported for OLEU-EE and HYT-EE, respectively (49). Our results show that both fluorescent AGEs have lower IC_50_ values than those reported (36, 49) for OLEU-EE, even with lower OLEU content in our extracts [43.4 mg OLEU/g vs. 93.9 mg OLEU/g (36)].

Figure 5 shows the antiglycant effect of OLEU-EE compared to HYT, OLEU, and AMIG standards, and for mean IC50 values of fluorescent AGEs (Figures 5A,B) and oxidated amino acids (Figures 5C,E), all have a very significant difference (^****^) with the OLEU-EE. OLEU-EE was 6.6, 4.4, and 3.9 times less effective than HYT, OLEU, and AMIG, respectively. Previous studies have shown that phenolic compounds can react with dicarbonylic molecules, such as methylglyoxal, using an electrophilic aromatic substitution, mechanisms that can explain the high reactivity of HYT (50, 51). Protein oxidative modifications such as DiTyr, N-Formyl Kyn, and Kyn can be produced as a consequence of glycoxidative and autoxidative reactions occurring during the generation of AGEs (52). Therefore, the inhibition of these modifications can also occur as a consequence of the inhibition of chemical pathways that give rise to AGEs.

The fact that HYT was 1.48% more effective than OLEU is in agreement with previous results that HYT-EE was more effective in inhibiting fluorescent AGEs compared to OLEU-EE (36). Although in the case of our OLEU-EE, the concentration of HYT is low (below the limit of quantification), which is why it is suggested that the main molecules responsible for the antiglycant activity would be the OLEU, OLEU aglycone, and luteolin glycosides according to the literature (30) and identification by UHPLC-MS. Although HYT shows greater antiglycant activity than OLEU, in this research, OLEU-EE was chosen as potential because obtaining it is considered a simple, cost-efficient, and environmentally friendly process. In addition, to obtain HYT-EE, several additional steps are necessary for the enzymatic or chemical conversion of OLEU to HYT. Also, to obtain HYT-EE in high concentration and purity, it is necessary to add purification steps. Another advantage of OLEU-EE over HYT-EE is the high stability of the first extract (>6 months corroborated by monitoring the OLEU signal by HPLC, results not included) and this is consistent with the literature (37). On the other hand, HYT-EE is unstable (53), probably due to the remarkable pharmacological and antioxidant activity of HYT (54), which is why several investigations have opted to stabilize this type of extract by encapsulation processes (55–57), which would further increase the cost of obtaining this potential functional ingredient.

## Conclusion

Three phenolic extraction techniques from olive mill leaves were evaluated using a combined Factorial Design to determine the parameters to obtain an OLEU-EE with a high content of TPC, AA, and OLEU. OLEU-EE #1 was selected using the HAE technique due to its high content of TPC (5,196 mg GA/100 g) and OLEU (4,345 mg of OLEU/100 g). In addition, HAE has the advantages of generating low solvent volume, and fast and high yield methods. HAE increases your extraction performance by combining high-speed impact force and intense shear. Therefore, HAE constitutes a simple and cost-effective method for the extraction of phenolics, and, in particular, OLEU from olive leaves. In addition, OLEU-EE #1 has a high antioxidant activity (57,867 μmol of TE/100 g) and an antiglycant effect, which demonstrates the action of OLEU-EE #1 on radical-mediated oxidative stress and inhibition of AGE (IC_50_ of 0.1899 and 0.1697 mg/mL for ^1^λ_EXC_ 325/λ_EM_ 440; ^2^λ_EXC_ 389/λ_EM_ 443, respectively), as well as in oxidative protein modifications (DiTyr, N-formyl Kyn, and Kyn were IC_50_ of 0.1852, 0.2044, and 0.1720 mg/mL, respectively). Therefore, the combination of a cost-effective extraction method with a low-cost raw material is an economical and environmentally friendly alternative for the recovery of this olive by-product.

## Data Availability Statement

The original contributions presented in the study are included in the article/Supplementary Material, further inquiries can be directed to the corresponding author/s.

## Author Contributions

The preparation of the material and chemical analysis were carried out by NM and KM (extracts for all of the DOE), analysis HPLC-MS by AB-E and analysis of antiglycating effect by NC and XP. The data collection and analysis was carried out by KM and FÁ. The review and editing of the manuscript were carried out by BC, the acquisition of funds and software was contributed by KM. The first draft of the manuscript was written by KM, and all authors commented on previous versions of the manuscript. All authors read and approved the final manuscript. All authors contributed to the conception and design of the study.

## Funding

This research was funded by Centro de Estudios en Alimentos Procesados-CEAP, ANID, and Regional Program R19A10001, Talca, Chile.

## Conflict of Interest

The authors declare that the research was conducted in the absence of any commercial or financial relationships that could be construed as a potential conflict of interest.

## Publisher's Note

All claims expressed in this article are solely those of the authors and do not necessarily represent those of their affiliated organizations, or those of the publisher, the editors and the reviewers. Any product that may be evaluated in this article, or claim that may be made by its manufacturer, is not guaranteed or endorsed by the publisher.
