# Supplementary material for: Oleuropein-Enriched Extract From Olive Mill Leaves by Homogenizer-Assisted Extraction and Its Antioxidant and Antiglycating Activities
Source: Front Nutr. 2022 Jun 23;9:895070. doi: 10.3389/fnut.2022.895070 (PMC9273007; doi:10.3389/fnut.2022.895070)
Supplement: Supplementary file 1 [file Data_Sheet_1.docx]

Supplementary Material

| 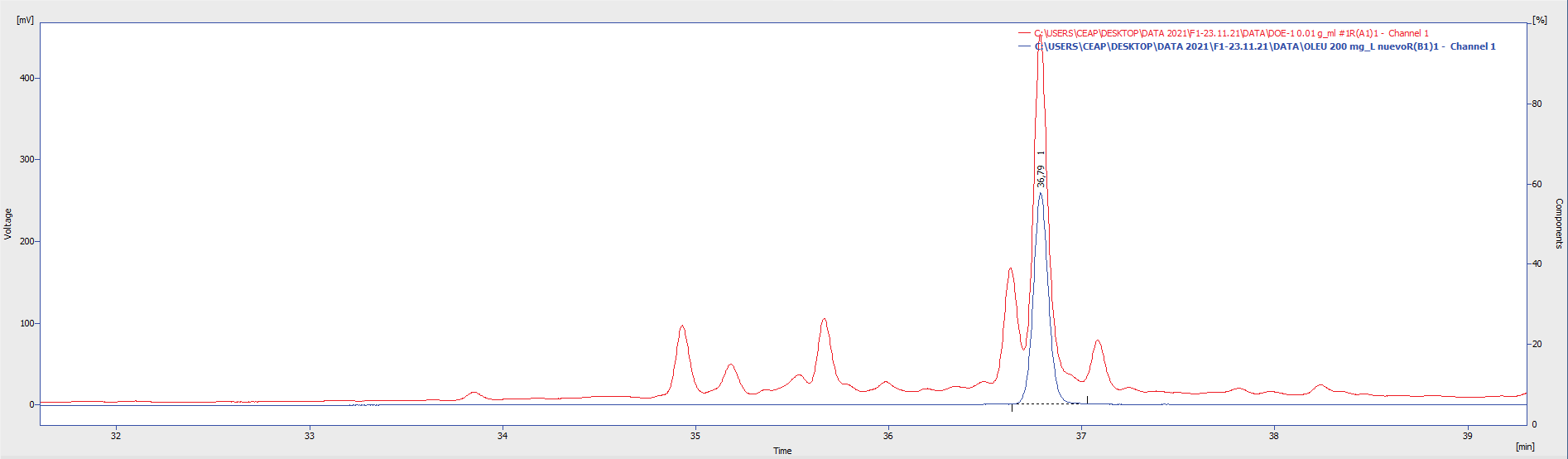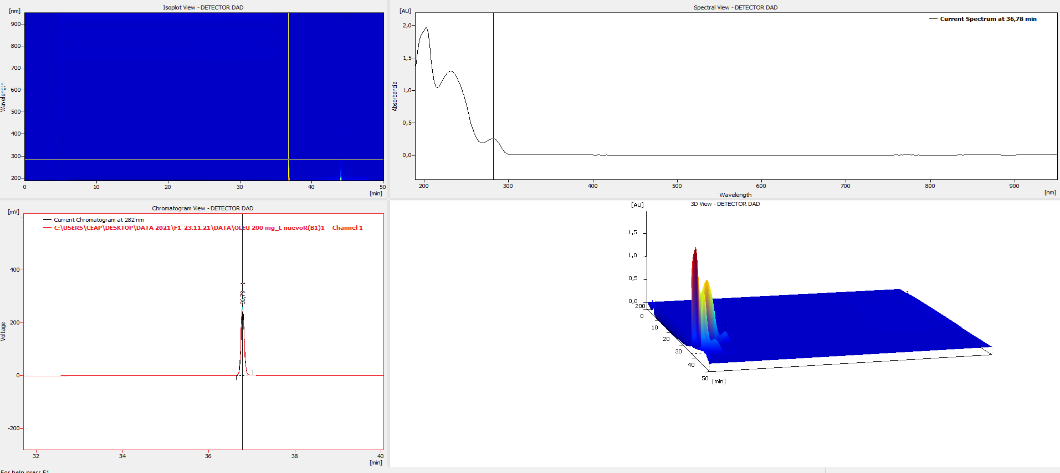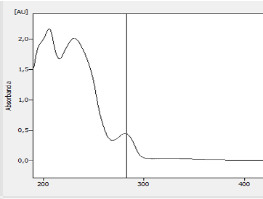 |
| --- |

**Figure S1:** Identification of oleuropein in OLEU-EE #1 analyzed by HPLC-DAD. Chromatogram and spectrogram to 280nm correspond to OLEU-EE #1 (line red) and OLEU 200 mg/L (line blue).


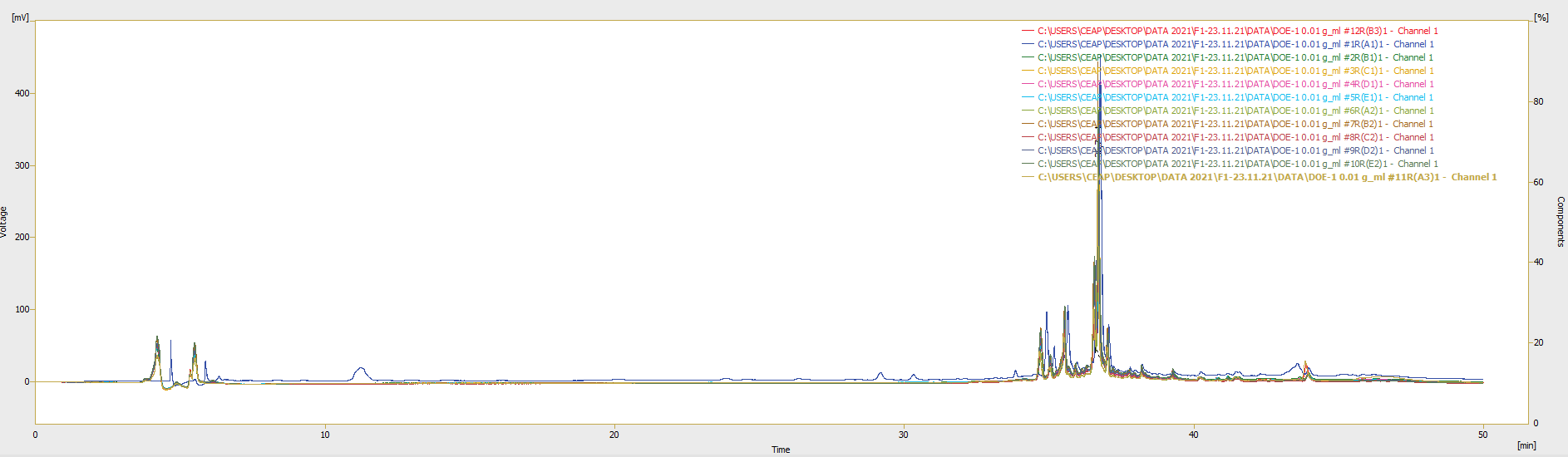


**Figure S2:** Chromatogram at 280nm of all OLEU-EE of the DOE-factorial analyzed by HPLC-DAD.


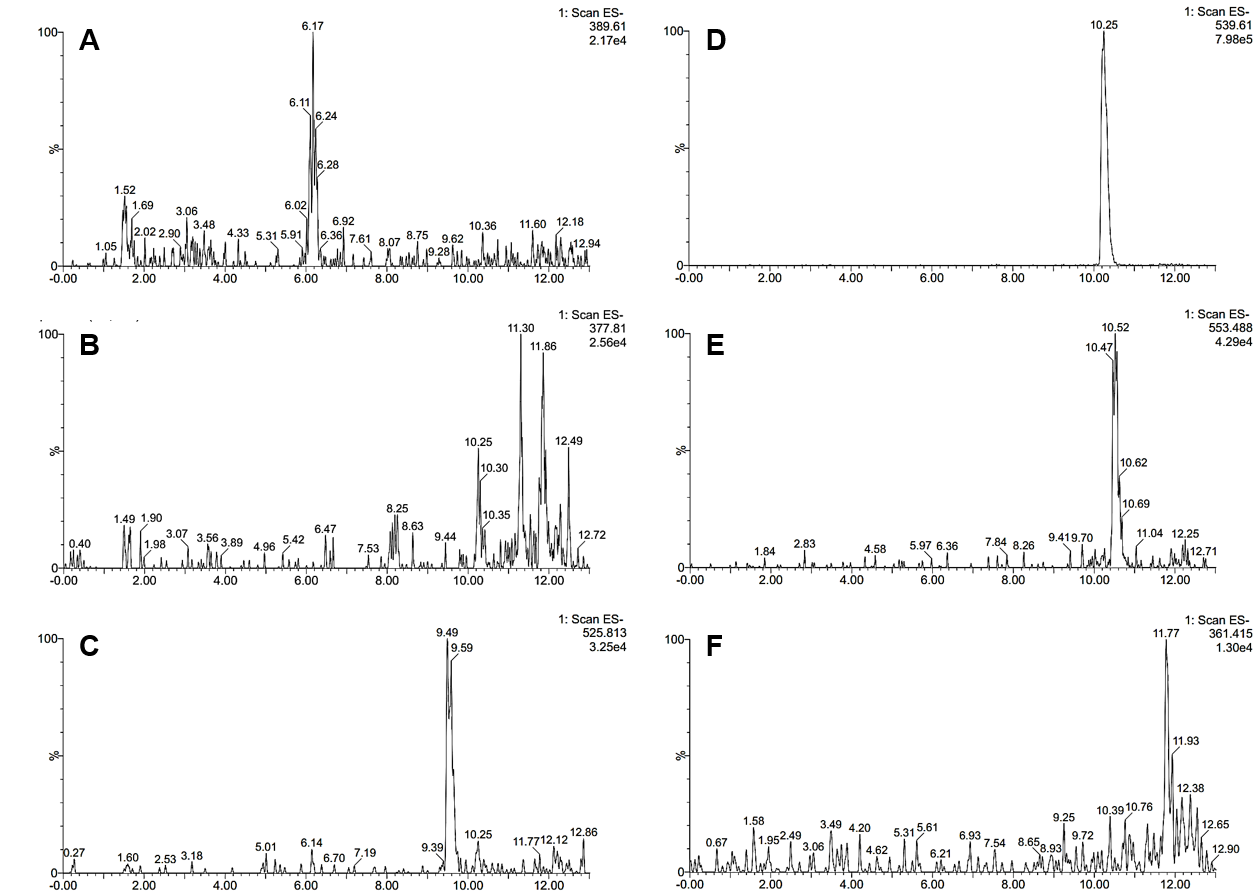


**Figure S3**. Extracted ion chromatograms (EIC) of the secoiridoids tentatively identified in the OLEU-EE #1 from olive mill leaves analyzed by UHPLC-MS: Oleoside (A), Oleuropein aglycon isomers (B), Demethyloleuropein (C), Oleuropein (D), Methyloleuropein (E), Ligstroside aglycon (F).


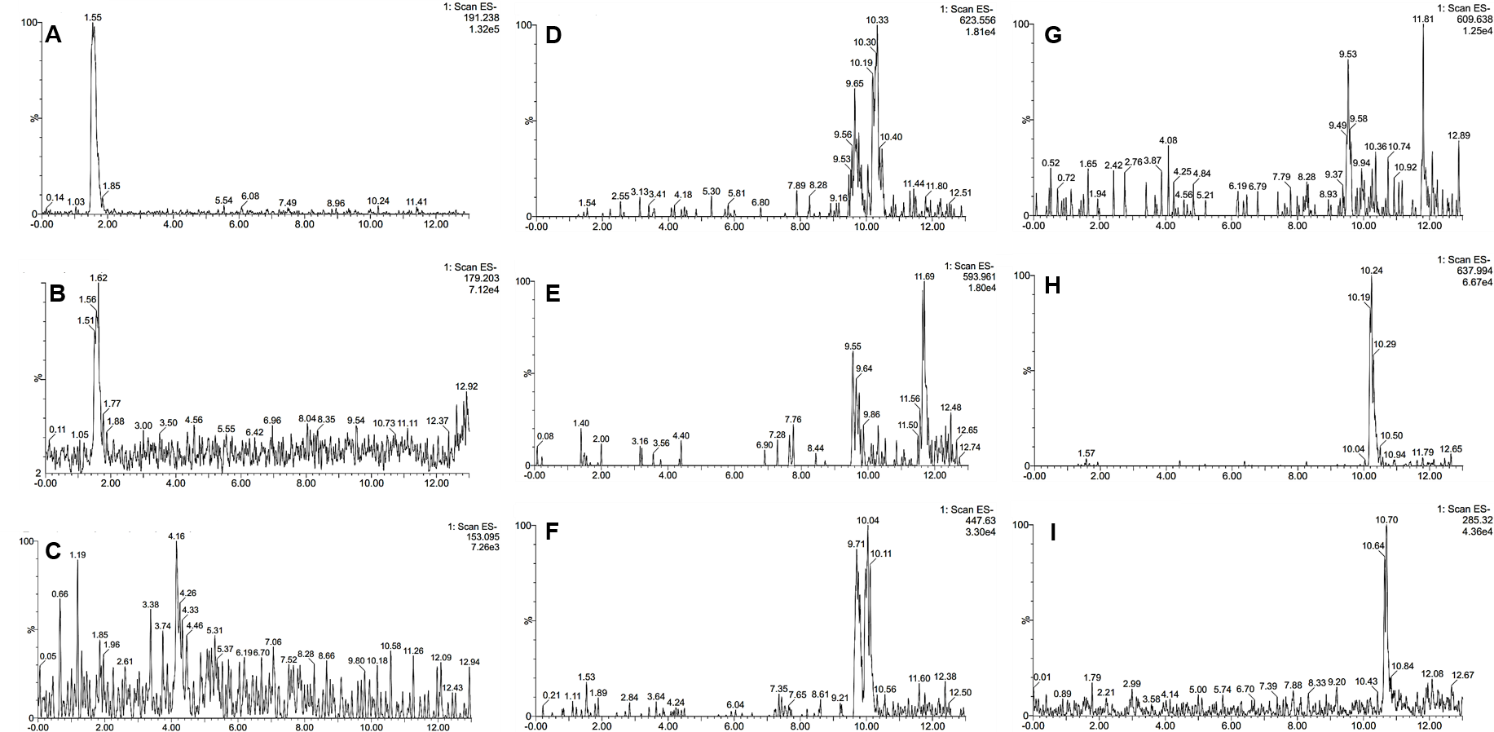


**Figure S4.** Extracted ion chromatograms (EIC) of the other compounds tentatively identified in the OLEU-EE #1 from olive mill leaves analyzed by UHPLC-MS: Quinic acid (A), Caffeic acid derivative (B), Hydroxytyrosol (C), Verbascoside (D), Luteolin rutinoside (E), luteolin hexosides (F), Rutin (G), Methylverbascoside (H), Luteolin (I).

**Table S1.** Statistical differences between the means of different samples for TPC, OLEU, and AA were determined by one-way ANOVA and Tukey test.

| **Groups** | **p value TPC** | **p value OLEU** | **p value AA** | **Groups** | **p value TPC** | **p value OLEU** | **p value AA** |
| --- | --- | --- | --- | --- | --- | --- | --- |
| 1 vs. 2 | <0,0001 | <0,0001 | <0,0001 | 4 vs. 8 | 0,0621 | <0,0001 | 0,9998 |
| 1 vs. 3 | 0,0001 | <0,0001 | 0,4382 | 4 vs. 9 | 0,0135 | <0,0001 | >0,9999 |
| 1 vs. 4 | 0,9604 | <0,0001 | 0,7671 | 4 vs. 10 | 0,9765 | <0,0001 | 0,2404 |
| 1 vs. 5 | <0,0001 | <0,0001 | 0,0071 | 4 vs. 11 | <0,0001 | <0,0001 | <0,0001 |
| 1 vs. 6 | <0,0001 | <0,0001 | <0,0001 | 4 vs. 12 | 0,6843 | <0,0001 | 0,9939 |
| 1 vs. 7 | 0,1274 | 0,9838 | 0,9762 | 5 vs. 6 | 0,0071 | 0,7006 | 0,0003 |
| 1 vs. 8 | 0,0027 | <0,0001 | 0,9890 | 5 vs. 7 | 0,0631 | <0,0001 | 0,0003 |
| 1 vs. 9 | 0,0005 | <0,0001 | 0,6692 | 5 vs. 8 | 0,8130 | <0,0001 | 0,0911 |
| 1 vs. 10 | >0,9999 | <0,0001 | 0,9979 | 5 vs. 9 | 0,9920 | <0,0001 | 0,4262 |
| 1 vs. 11 | <0,0001 | <0,0001 | <0,0001 | 5 vs. 10 | <0,0001 | <0,0001 | 0,0008 |
| 1 vs. 12 | 0,0821 | <0,0001 | 0,1936 | 5 vs. 11 | 0,2395 | <0,0001 | 0,0101 |
| 2 vs. 3 | >0,9999 | <0,0001 | 0,0021 | 5 vs. 12 | 0,0993 | 0,3522 | 0,9156 |
| 2 vs. 4 | 0,0024 | <0,0001 | 0,0006 | 6 vs. 7 | <0,0001 | <0,0001 | <0,0001 |
| 2 vs. 5 | >0,9999 | <0,0001 | 0,1936 | 6 vs. 8 | <0,0001 | <0,0001 | <0,0001 |
| 2 vs. 6 | 0,0030 | 0,0001 | 0,2084 | 6 vs. 9 | 0,0005 | <0,0001 | <0,0001 |
| 2 vs. 7 | 0,1318 | <0,0001 | <0,0001 | 6 vs. 10 | <0,0001 | <0,0001 | <0,0001 |
| 2 vs. 8 | 0,9488 | <0,0001 | <0,0001 | 6 vs. 11 | 0,8699 | <0,0001 | 0,9284 |
| 2 vs. 9 | 0,9998 | <0,0001 | 0,0008 | 6 vs. 12 | <0,0001 | 0,0059 | <0,0001 |
| 2 vs. 10 | 0,0001 | <0,0001 | <0,0001 | 7 vs. 8 | 0,8442 | <0,0001 | 0,4382 |
| 2 vs. 11 | 0,1231 | <0,0001 | 0,9546 | 7 vs. 9 | 0,4416 | <0,0001 | 0,0950 |
| 2 vs. 12 | 0,1982 | <0,0001 | 0,0071 | 7 vs. 10 | 0,1537 | <0,0001 | >0,9999 |
| 3 vs. 4 | 0,0031 | <0,0001 | >0,9999 | 7 vs. 11 | <0,0001 | <0,0001 | <0,0001 |
| 3 vs. 5 | >0,9999 | <0,0001 | 0,6564 | 7 vs. 12 | >0,9999 | <0,0001 | 0,0134 |
| 3 vs. 6 | 0,0023 | <0,0001 | <0,0001 | 8 vs. 9 | 0,9999 | <0,0001 | 0,9986 |
| 3 vs. 7 | 0,1642 | <0,0001 | 0,0433 | 8 vs. 10 | 0,0034 | 0,1846 | 0,6434 |
| 3 vs. 8 | 0,9712 | >0,9999 | 0,9762 | 8 vs. 11 | 0,0053 | <0,0001 | <0,0001 |
| 3 vs. 9 | >0,9999 | <0,0001 | >0,9999 | 8 vs. 12 | 0,9278 | <0,0001 | 0,8111 |
| 3 vs. 10 | 0,0002 | 0,3460 | 0,0873 | 9 vs. 10 | 0,0007 | <0,0001 | 0,1796 |
| 3 vs. 11 | 0,0978 | <0,0001 | <0,0001 | 9 vs. 11 | 0,0262 | 0,2772 | <0,0001 |
| 3 vs. 12 | 0,2426 | <0,0001 | >0,9999 | 9 vs. 12 | 0,5769 | <0,0001 | 0,9986 |
| 4 vs. 5 | 0,0010 | <0,0001 | 0,3356 | 10 vs. 11 | <0,0001 | <0,0001 | <0,0001 |
| 4 vs. 6 | <0,0001 | <0,0001 | <0,0001 | 10 vs. 12 | 0,1003 | <0,0001 | 0,0287 |
| 4 vs. 7 | 0,8091 | <0,0001 | 0,1317 | 11 vs. 12 | 0,0001 | <0,0001 | 0,0003 |

**Table S2.** Statistical differences between the means of different samples for antiglycating effect were determined by one-way ANOVA and Tukey test.

| **Group 1** | **p value AGE^1^** | **p value AGE^2^** | **p value Di-Tyr** | **p value N-formyl Kyn** | **p value Kyn** |
| --- | --- | --- | --- | --- | --- |
| OLEU-EE vs. HYT | <0,0001 | <0,0001 | <0,0001 | <0,0001 | <0,0001 |
| OLEU-EE vs. OLEU | <0,0001 | <0,0001 | <0,0001 | <0,0001 | <0,0001 |
| OLEU-EE vs. AMIG | <0,0001 | <0,0001 | <0,0001 | <0,0001 | <0,0001 |
| HYT vs. OLEU | 0,0145 | 0,2373 | 0,0037 | 0,0024 | 0,5924 |
| HYT vs. AMIG | 0,0014 | 0,0029 | 0,0011 | 0,0002 | 0,0087 |
| OLEU vs. AMIG | 0,2974 | 0,0423 | 0,7127 | 0,1399 | 0,0498 |
